# A Case of Pancytopenia Caused by an Unusual Habit of Eating Pasta

**DOI:** 10.7759/cureus.6028

**Published:** 2019-10-30

**Authors:** Salim R Surani, Munish Sharma

**Affiliations:** 1 Internal Medicine, Texas A&M Health Science Center, Temple, USA; 2 Internal Medicine, Corpus Christi Medical Center, Corpus Christi, USA

**Keywords:** pancytopenia, anemia, vitamin b12, folate, pasta

## Abstract

Pure nutritional deficiency of folate and vitamin B12 is very unusual in developed countries, where most of the food items are fortified with essential vitamins and minerals. We hereby present a case of a middle-aged lady who declined to eat any other food items and survived only on pasta and sea salt for two years before presenting to the emergency department with pancytopenia.

## Introduction

Vitamin B12 and folate are water-soluble B vitamins. They are essential for hematopoiesis. Deficiency of these vitamins causes megaloblastic anemia characterized by impairment of nucleic acid metabolism, asynchronous nuclear-cytoplasmic ratio and nuclear abnormalities in the erythroid and myeloid lineage [1]. Due to the fortification of food items with folate, isolated deficiency of folic acid due to insufficient nutrition is rare in the United States of America (USA). Most of the people in the USA receive adequate amounts of folic acid [2]. Mean erythrocyte folate concentration among adults in the USA ranges from 216 to 398 ng/ml, which is considered as adequate folate supplementation [3]. In contrast, vitamin B12 deficiencies are still prevalent mainly due to malabsorption [4]. It can affect 1.5% to 15% of the general population but it is still low compared to other nations such as India where it can be prevalent in as high as 70% adults [5]. Vitamin B12 deficiency solely due to poor nutritional intake is rarely encountered in routine clinical practice, especially in developed countries.

## Case presentation

**Figure 1:**
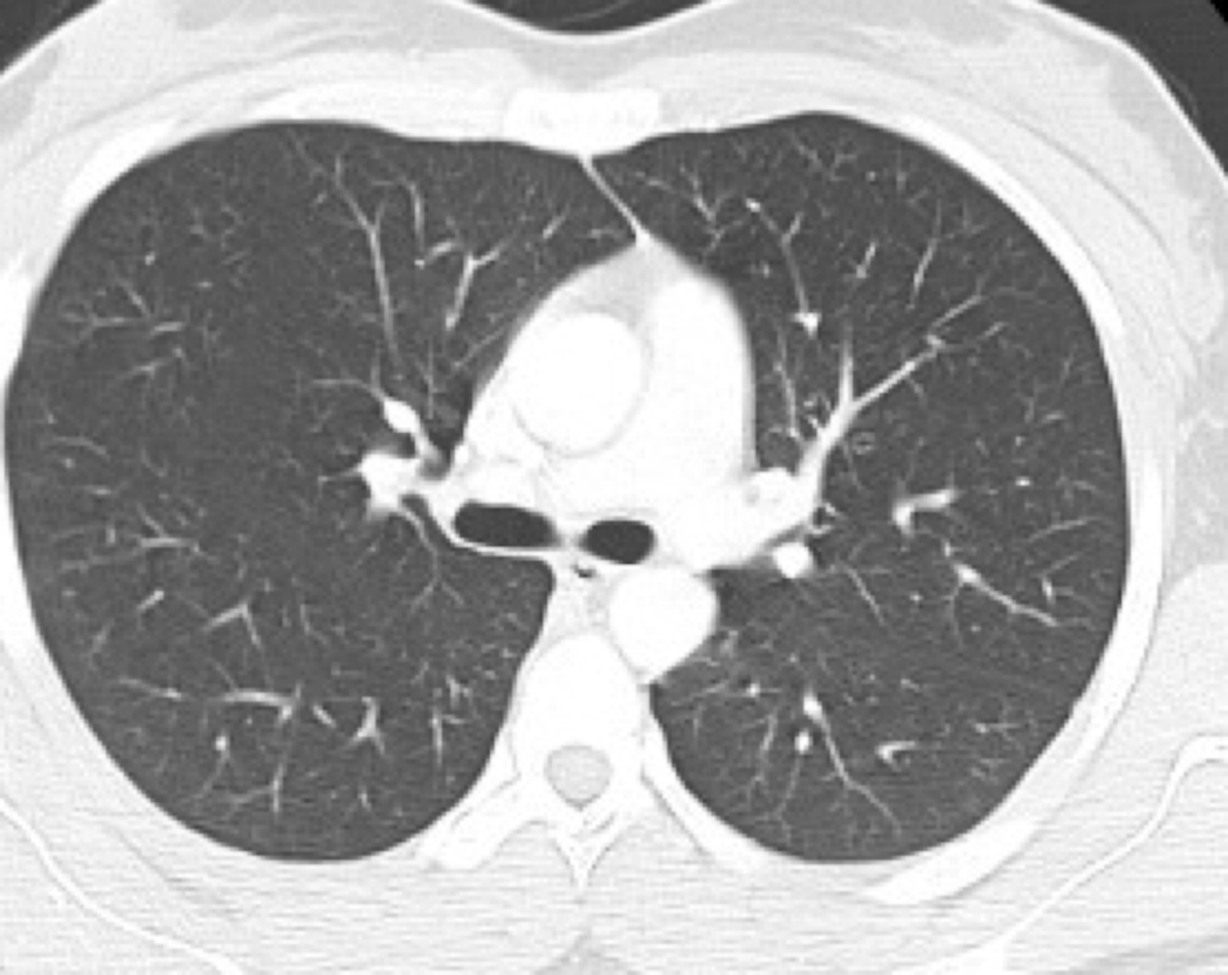
Computed tomography (CT) of the chest did not reveal any abnormality

**Figure 2:**
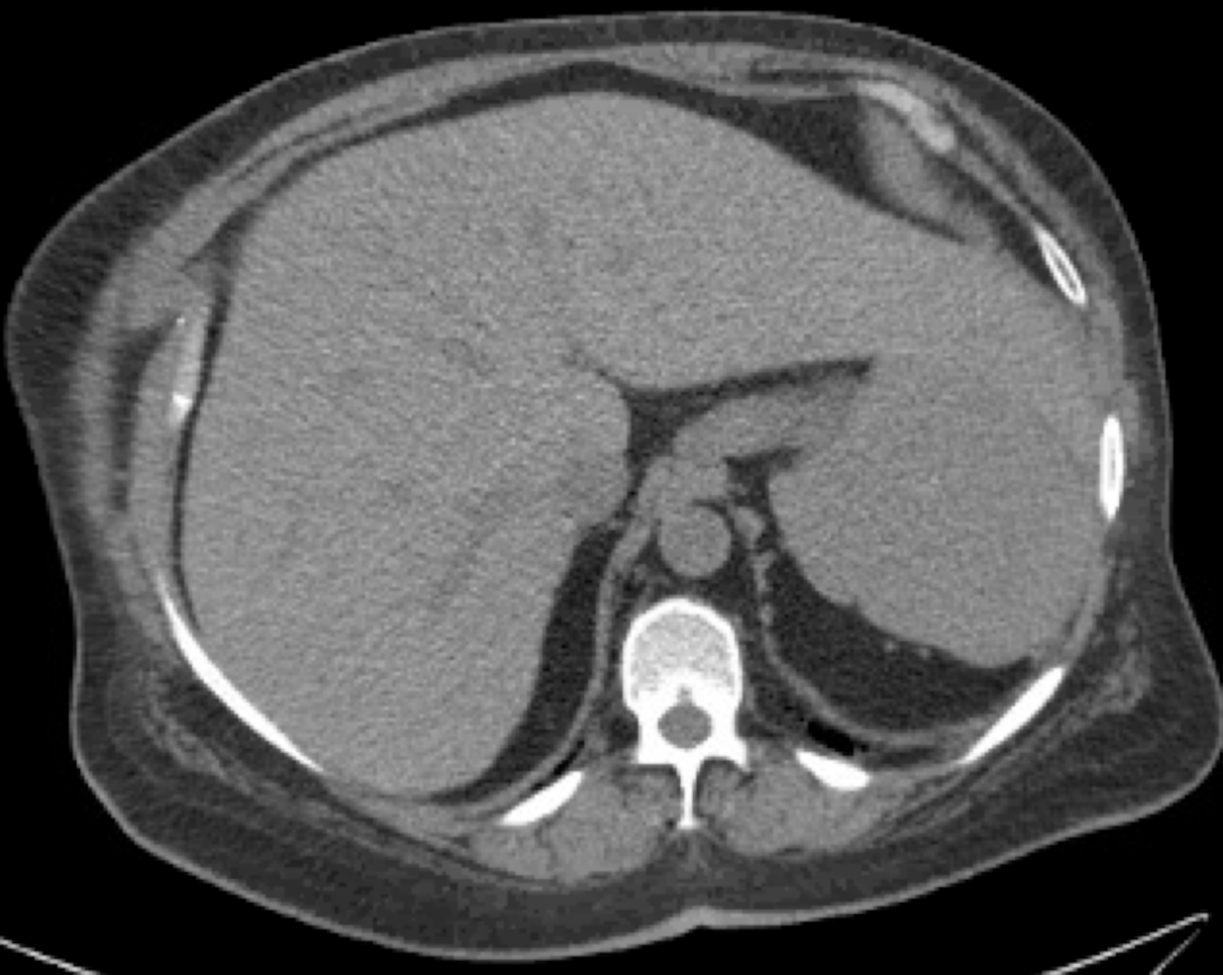
Computed tomography (CT) of the abdomen did not reveal any abnormality

A 40-year-old Caucasian female with a history of anxiety presented to our emergency department (ED) with gradual worsening weakness, intermittent blurry vision, and intermittent paresthesia over three weeks. On examination, her blood pressure was 120/69 mm Hg, respiratory rate 16/minute, temperature 98.30 F, heart rate 128 beats/minute, and oxygen saturation 99% on room air. The patient's appearance was frail with a body mass index (BMI) of 16.5 kg/m2. She had conjunctival pallor. Her mental status, all 12 cranial nerves examination, motor system, deep tendon reflexes, sensory system examination, coordination, station and gait examination did not reveal any abnormality. Remaining physical examinations were also unremarkable. Total white blood count (WBC) was 1100/microliter of blood (range: 4000-1100), hemoglobin 3.7 gram/deciliter, mean corpuscular volume 105.7 femtoliter (range: 80-96) and platelet count was 29000/microliter of blood (range: 150,000-450,000). Reticulocyte index was less than 2% (range: 0.5%-2.5%). She denied any recent bleeding, hematemesis, hematuria, dark stools or menorrhagia. Her blood urea nitrogen was 10 mg/dl (range: 7-20 mg/dl), serum creatinine 0.5 mg/dl (range: 0.6-1.2mg/dl), total bilirubin 0.9 mg/dl (range: 0.1-1.2 mg/dl), aspartate aminotransferase 13 units/liter (range: 10-40 units/liter), alanine aminotransferase 18 units/liter (range: 7-56 units/liter), alkaline phosphatase 14 units/liter (range: 20-40 units/liter), thyroid-stimulating hormone 2.3 milli-international units/liter (range: 0.4-4.0 milli-international units/liter) and serum copper level was 82 mg/dl (range: 70-140 mg/dl). There was no recent exposure to new medications. The patient had no family history of blood dyscrasias and also no history of fever, weight loss, night sweats or loss of appetite. Computed tomography (CT) of the chest did not reveal an abnormality (Figure 1). CT of the abdomen and pelvis did not indicate any lymphoproliferative disorder or splenomegaly (Figure 2).

Liver ultrasound showed normal liver echotexture. Human immunodeficiency virus (HIV) screening, hepatitis B and C panel, and urine toxicology screen were negative. On careful review of history, she revealed that she has "food allergies" and thus had been eating only pasta and sea salt for the past two years. She had never seen an allergist and had not undergone any testing that would truly establish her suspicion of food allergies. She had not visited a primary care physician for years leading to this visit. On further testing, serum folate level was <2 ng/ml (normal range: >7 ng/ml) and vitamin B12 level was 16.3 ng/ml (range: 200-900 ng/ml). She was transfused four units of packed red blood cells. She was given cyanocobalamin intramuscular injection 1000 mcg daily for one week, then weekly for one month followed by a monthly injection of 1000 mcg. Flow cytometry showed relative neutropenia with left-shifted maturation and 0.1% myeloid blasts. There was no immune phenotypic evidence of a B or T cell lymphoproliferative disorder. The patient's hemoglobin improved to 10.3 gm/dl after four days (Table 1). She was also recommended to follow up with a psychiatrist and nutritionist on discharge from the hospital. Her complete blood count showed a complete resolution of pancytopenia at two months follow up. Her vitamin B12 and folate level also normalized (Table 2).

**Table 1:** Basic complete blood count values on admission and on discharge

	Day of admission	4 days after admission
White blood cell count	1.1 per microliter of blood	2 per microliter of blood
Red blood cell count	2.80 million cells/ microliter	3.6 million cells/ microliter
Hematocrit	11.1 %	29.3 %
Hemoglobin	3.7 gram/deciliter	10.3 gram/deciliter (post-transfusion)
Mean corpuscular volume (MCV)	105.7 femtoliter/red cell	100.2 femtoliter/red cell
Mean corpuscular hemoglobin (MCH)	32.8 picograms per cell	33.4 picograms per cell
Mean corpuscular hemoglobin concentration (MCHC)	34.9 grams per deciliter	33.7 grams per deciliter
Red cell distribution width (RDW)	18.5 %	17.7 %
Platelet count	29 per microliter of blood	90 per microliter of blood

**Table 2:** Improvement in vitamin B12 and folate level after treatment

	Day of admission	After 2 months
Folate	<2 nano gram/milliliter	7.2 nano gram/milliliter
Vitamin B12	16.3 nano gram/milliliter	> 2000 nano gram/milliliter

## Discussion

Folate deficiency in isolation is uncommon. It generally co-exists with other nutritional deficiencies. In a study from a developed country, folate deficiency was found to have a prevalence of 0.06% only as compared to 7% to 79% in countries with low-income economies [6]. People at risk of folate deficiency include those with alcohol use disorder, women of childbearing age, pregnancy, and those with malabsorptive syndrome and methylenetetrahydrofolate reductase (MTHFR) polymorphism [7-8]. Folate is naturally present in green leafy vegetables, meat, poultry, fruits, and eggs. Recommended daily dietary allowances for adults is 400 mcg dietary folate equivalent (DFEs) [9]. Factors predisposing to vitamin B12 deficiency include atrophic gastritis, pernicious anemia, gastrointestinal disorders such as Chron's disease, celiac disease, strict vegetarians, pregnant and lactating women [10]. Vitamin B12 is abundant in natural animal products but is absent in plant foods. Recommended daily intake for individuals aged 14 and older is 2.4 mcg [10]. As per the data derived from the U.S. Department of Agriculture (2019), brands of pasta enriched with vitamins contain 102 micrograms of folate while whole wheat pasta contains 7 micrograms of folate per one cup of serving equivalent to 140 grams. In both of these forms, vitamin B12 was reported to be none [11]. Vitamin B12 and/or folate deficiency should be suspected in individuals with megaloblastic anemia or pancytopenia. Patients can also have gastrointestinal symptoms such as glossitis, oral ulceration or symptoms of underlying conditions such as Crohn's disease. The patient may present with neuropsychiatric manifestations in both vitamin B12 and folate deficiency. Most prominent deficits include gait disturbances, paresthesia, dementia, psychosis and optic atrophy [4]. Laboratory diagnoses include a complete blood count and vitamin B12/folate level. Additional testing with homocysteine, methylmalonic acid, and autoantibodies to intrinsic factor may be required if vitamin B12 level is borderline. Red blood cell folate may be required if the level of folate is borderline [12]. 

Since the total body stores of vitamin B12 is equivalent to 3 to 5 mg, it can last for up to five to 10 years before manifestations of vitamin B12 is seen clinically. In contrast, folate deficiency can manifest over weeks to months depending on the baseline body stores [13]. Our patient was eating only pasta and sea salt for two years. She was afraid of eating any vegetables, fruit, meat, egg or dairy products. She never had any proven food allergies. It was astonishing to see how she never sought any medical attention until she was critically ill. 

For patients with concerning symptoms of anemia or neuropsychiatric manifestations, parenteral therapy with injections (vitamin B12 1,000 µg subcutaneous or intramuscular) every week for one month followed by the same dose every month is given until the deficit is corrected. Since our patient had neurologic manifestations and biochemical evidence of severe vitamin B12 deficiency, we treated her with a parenteral form of vitamin B12. Oral vitamin B12 at a dose of 1000 to 2000 micrograms per day can be an effective alternative if adherence or malabsorption is not a concern [14]. Folate deficiency is treated with 1 to 5 mg of folic acid supplement daily until hematological values improve and the recovery of the clinical symptoms is seen [15]. 

## Conclusions

Pure nutritional deficiency of folate and vitamin B12 in developed countries is rare, where most of the food items are fortified with essential vitamins and minerals. Extreme irrational fear of "food allergies" and the unwillingness to visit physicians led to late detection of severe pancytopenia due to vitamin B12 and folate deficiency in our patient. Careful history taking and basic laboratory testing are sufficient to diagnose and treat this infrequently encountered cause of pancytopenia. Also, it provokes us to consider the need for the fortification of routinely consumed food items. Further clinical studies are needed to re-evaluate the fortification needs of dietary products.
